# A novel knowledge extraction method based on deep learning in fruit domain

**DOI:** 10.1038/s41598-022-26116-y

**Published:** 2022-12-13

**Authors:** Xinliang Liu, Lei Ma, Tingyu Mao, Mengqi Zhang, Yong Li, Yanzhao Ren

**Affiliations:** 1 School of E-business and Logistics, Beijing Technology and Business University, Beijing, 100048 China; 2 National Engineering Laboratory for Agri-product Quality Traceability, Beijing Technology and Business University, Beijing, 100048 China; 3 School of Computer Science and Engineering, Beijing Technology and Business University, Beijing, 100048 China

**Keywords:** Computer science, Information technology

## Abstract

Knowledge extraction aims to identify entities and extract relations between them from unstructured text, which are in the form of triplets. Analysis of the fruit nutrition domain corpus revealed many overlapping triplets, that is, multiple correspondences between a subject and multiple objects or the same subject and object. The current relevant methods mainly target the extraction of ordinary triplets, which cannot accurately identify overlapping triplets. To solve this problem, a deep learning based model for overlapping triplet extraction is proposed in this study. The relation is modeled as a function that maps a subject to an object. The hybrid information of the subject is entered into the relation-object extraction model to detect the object and relation. The experimental results show this model outperforms existing extraction models and achieves state-of-the-art performance on the manually labeled fruit nutrition domain dataset. In terms of application value, the proposed work can obtain a high-quality and structured fruit nutrition knowledge base, which provides application fundamentals for downstream applications of nutrition matching.

## Introduction

Data from “Big data about nutrition” show that public concern for fruit nutrition-related information is growing^1^. Reasonable nutrition matching is an important foundation for public health. At present, many platforms on the Internet contain massive amounts of information related to fruit nutrition. However, such information is complicated, uneven and multi-sourced and heterogeneous, raising multiple obstacles to the access of fruit nutrition knowledge. Moreover, fruit nutrition information has the characteristics of professionalism and standardization. Therefore, how to extract fruit nutrition knowledge automatically and accurately to support intelligent recommendation tasks in fruit nutrition matching has become the focus of current research.

The triplet extraction method is a key technique for knowledge extraction^2^, which can automatically extract fruit nutrition knowledge from unstructured text. There is a triplet (Bananas,rich in,protein) in the sentence “Bananas are rich in protein and other nutrients”. The choices of the triplet extraction method are highly correlated with the structural characteristics of the corpus. Triplet extraction consists of two processes: identifying the content, types, and boundaries of entities from an unstructured corpus and classifying the semantic relations between these entities. Recent advances include two mainstream extraction methods: the pipeline extraction methods^3^ and joint extraction methods^4–6^. The pipeline approach employs named entity recognition^7–10^ and relation classification^11^ as two separate processes. Conversely, joint extraction method treats knowledge extraction as a single task optimized in a unified model. Miwa et al. proposed a new end-to-end model to extract the relations between entities in word sequences and dependency tree structures. This approach shared the representation parameters of entities and relations in a single mode. But it ignored long-range dependencies between entity labels^12^. To overcome this problem, Zheng et al.^13^ transformed joint extraction into sequence labeling. However, Zheng’s model cannot assign multiple relations to a token. Thus it cannot solve the overlapping problem, which is currently a hot research topic in the field of knowledge extraction. The triplets obtained from the corpus of fruit nutrition have overlapping characteristics, including SingleEntityOverlap and EntityPairOverlap. SEO means that a subject corresponds to two or more objects, and EPO means there are at least two relations between a pair of subject and object as shown in Table 1.

**Table 1:** Examples of the Normal, SingleEntityOverlap (SEO), and EntityPairOverlap (EPO) cases. The overlapping entities are marked in blue.

Types	Sentences	Triplets
Normal	Kiwifruit belongs to the family Actinidiaceae.	(Kiwifruit,belong to,Actinidiaceae)
SPO	Kiwifruit is rich in vitamin C, grape acid and other nutrients.	(Kiwifruit,rich in,vitamin C)
		( Kiwifruit,rich in,grape acid)
EPO	Kiwifruit is mainly processed and produced in Shaanxi.	( Kiwifruit,origin,Shaanxi)
		( Kiwifruit,process,Shaanxi)

To overcome the overlapping problem, Liu et al. and Dai et al. applied the entity position information and context-dependent feature information to different layers of the model to assist in relation classification^14,15^. Yet, they neglected label features, which made the efficiency of the relation extraction task insignificant. Wang et al. tried table-sequence encoding, Miwa et al. used table representation, and Qin et al. designed entity indicators to obtain rich semantic information from sentences^16–18^. Entity indicators performed well in named entity recognition but had low accuracy in relation extraction. Therefore, improving the accuracy of extracting overlapping triplets while maintaining the precision of extracting all triplets is the goal of this study. To this end, we propose a novel extraction model for obtaining overlapping triplets from the Chinese corpus in the fruit nutrition domain. The model contains two sub-algorithms: subject identification algorithm and relation-object detection algorithm. The main contributions of this study are as follows:

1. This study provides a subject-oriented overlapping triplet extraction model (SOOT), which models the fruit nutrition the task of entity and relation extraction as a function to handle the extraction of overlapping triplets.
2. We consider the impact of information dependency between the sub-models and add label information of the subject and character to the object recognition task in the extraction process.
3. We employ multiple binary taggers as relations. Thus, the model can determine the object that corresponds to the subject under different relations, which reduces the noise from irrelevant entities.
4. Analysis of the publicly available New York Times (NYT) dataset and a self-built fruit nutrition domain text dataset (FND) demonstrates that SOOT markedly outperforms state-of-the-art joint methods, achieving 2.5% and 4.8% absolute F1-value gains in two datasets, respectively.

## Related works

Conventional extraction methods divide triplet extraction task into two steps: named entity identification, which identifies all possible entities including their contents, boundaries and types from the input sequence; relation classification, which classifies the semantic relations between entity pairs. Such a framework makes the task easy to conduct. However, the limitation of this pipeline approach is that it ignores the dependency between the two subtasks and is prone to error propagation.

Recent research has tended to perform joint extraction of entities and relations in the same model, and joint extraction models perform well. Initially, scholars usually divide joint extraction models into three layers: the input layer, where the input words or characters are vectorized using pre-trained language models, such as bidirectional encoder representation from transformers (BERT)^19^, word2vec^20^, and fastText^21^ to obtain a vector representation; the neural network layer, where a convolutional neural network (CNN)^22^, recurrent neural network (RNN)^23^, long short-term memory (LSTM)^24^ and other variants of neural networks^25^ are tried to obtain the potential semantic representation of the input vector; and the output layer, which employs SoftMax and sigmoid functions for relation classification. Although these models perform well in triplet extraction, they are applicable when the input sequence contains only ordinary triplets. They cannot extract overlapping triplets.

In studies related to the extraction task of overlapping triplets, Liu et al. merged the encoding of lexical features, entity features, and position features extracted from text and then entered them into a dynamic LSTM to identify the overlapping relations between multiple entities in a sentence^14^. Bekoulis et al. modeled the extraction task as a multi-head selection problem by first identifying all entities in a sentence and then tagging the entities with BIO tags. The label features and hidden information were entered into the classifier to obtain the relation. The improvement in the F1 values of this model for the overlapping triplet problem was not very significant^26^. Wang et al. designed three token links (entity head to entity tail, subject head to object head, and subject tail to object tail) to represent triplets and then proceeded a SoftMax function to classify the relations of the linked entity pairs. This model employed a single-stage process to predict overlapping relations based on information from entity pairs^27^. Hang et al. provided a BERT-based parameter-sharing layer to capture the features of entities and relations and then combined Source-Target BERT and overlapping relation extraction model (OREM) to generate an unlimited number of relational triplets. Admittedly, these authors ignored the importance of label features for relation discrimination^28^. Zeng et al. introduced a multi-task framework that added a sequence-labeling layer. It allowed the model to predict entities with multiple characters^29^. Nevertheless, this model did not have high extraction accuracy in Chinese dataset. In contrast to previous models, Wei et al. proposed a new cascading binary tagging framework using a two-layer tagger (CASREL) to extract overlapping triplets. This tagger first identified all possible subjects in a sentence and then entered the subjects into a relational model to determine the corresponding objects based on predefined relations. The subject tagger classified the output of the BERT encoder through the fully connected layer. But it did not adequately capture other features of the entity. Thus, this model was unable to consider the dependency features of the labels for relational mapping^30^.

Although the existing models perform well, most of them ignore the link between entity identification and relationship extraction. This leads to poor F1 value of the model in extracting overlapping triplets. The analysis of existing models demonstrates that BERT outperforms other pre-trained language models. Moreover, BERT can obtain deeper and richer semantic information, such as contextual information, position encoding information, character information and other feature information. Such information is crucial for knowledge extraction tasks. Therefore, BERT is introduced as the encoder in most studies. Conditional random field (CRF), is usually added after the encoder. Because the labels output by the encoder may not be entirely correct, CRF can learn to transfer scores between adjacent labels to obtain the correct label.

Based on the research described above, this study proposes an enhanced subject-oriented approach for Chinese text in the fruit nutrition domain. We apply the Chinese pre-trained BERT model (The following section is denoted with BERT) known as “BERT-wwm-ext” to generate the raw token representations. The model can be further improved by using a properly pre-trained language model. Then, we try the CRF to identify the type and contextual features of the subjects. Finally, we employ fusion label of the subject and character as the input feature for the object detection task. SOOT leverages binary taggers to detect the position of the objects. The results show that SOOT significantly outperforms baseline approaches.

## Methods

### Task definition

We first define the problem of the joint extraction of overlapping triplets. Given a sentence with n words, ${W}=({w}_1,{w}_2,\ldots {w}_n)$, and a set of predefined relations ${R}=({r}_1,{r}_2,\ldots {r}_n)$, it aims to extract one or more triplets from normal, EPO, and SEO sentences.

1 $$\begin{aligned} Y=\{(s,r,o)|(s,o)\in E,r\in R\} \end{aligned}$$

where *s* represents the subject in the triplet, *o* represents the object in the triplet, *r* is the relation between *s* and *o*, and *E* is a set of candidate entities.

### Model function

A subject-oriented overlapping triplet extraction model is presented to extract overlapping triplets in Chinese fruit nutrition in this study. The SOOT model maps the subject to the object, treating the relation as a function. It is defined as Eq. (2):

2 $$\begin{aligned} f_r(s)=o\xrightarrow {}T(s,r,o) \end{aligned}$$

where T is the collection of triplets.

### Model design

**Figure 1:**
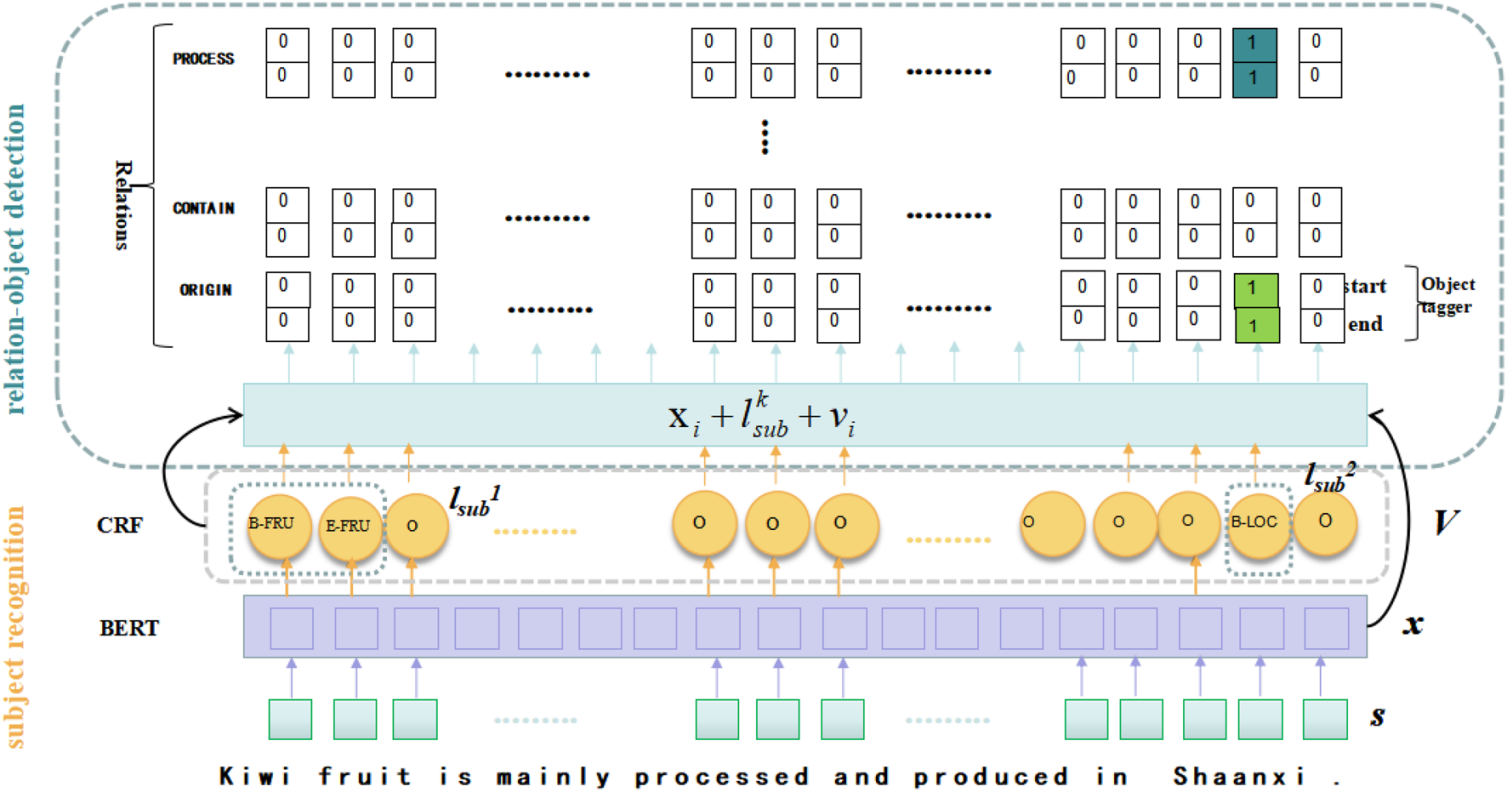
Subject-oriented overlapping triplets extraction model. This model takes the sentence “Kiwifruit is mainly processed and produced in Shaanxi.” as input, and the output triplets are (kiwifruit,origin,Shaanxi) and (kiwifruit,process,Shaanxi).

The framework of SOOT consists of three main parts: the encoder, subject recognition model, and relation-object detection model. Fig. 1 shows the general framework of SOOT, which comprises three steps:

- *Step 1* BERT is introduced as an encoder to obtain the semantic information of tokens in sentences and pass it to two sub-models.
- *Step 2* The subject recognition model applies CRF for named entity recognition and outputs all possible subjects in the sentence and the corresponding labels.
- *Step 3* The relation-object detection model adopts object taggers to extract objects corresponding to subjects under particular relations.
- We describe the details of each part in the following subsections.

- *Encoders*
  The two subtasks of this model share the same BERT encoder, which extracts feature information from the input sentences and provides it to the two subsequent sub-extraction models.
  Given a sentence input *S*=(${s}_1$,${s}_2$,...${s}_n$) containing *n* characters, *S* is entered into the BERT pre-trained language model to obtain a vector representation of the sentence $\varvec{X}$=($\varvec{x}_1$,$\varvec{x}_2$,...$\varvec{x}_n$), where $\varvec{x}_i$ is the character vector of the $i_{th}$ word.
- *Subject recognition algorithm*
  **CRF Layer** The subject recognition algorithm employs CRF model to detect the class of entities and their boundaries. The output $\varvec{X}$ of the BERT encoder is entered into the CRF model to obtain the label sequence *V*=(${v}_1$,${v}_2$,...${v}_n$). The entities and their types are obtained by identifying the tag sequence. The calculation formulae are defined as Eqs. (3) and (4):
  3 $$\begin{aligned} S(x,y)= & {} \sum _{i=0}^{n}(A_{y_{i},y_{i+1}})+\sum _{i=0}^{n}(P_{i,i+1}) \end{aligned}$$
  4 $$\begin{aligned} y^*= & {} argmaxS(x,y) \end{aligned}$$
  where $\varvec{x}$ is the character vector of the sentence, *i* is the number of tags, and $\varvec{A}$ is the transfer matrix; $A_{yi,yi+1}$ denotes the transfer score from the $y_{i}$ tag to the $y_{i+1}$ tag and ${y^*}$ denotes the sequence with the highest probability value calculated.
  **Label feature** We apply the markers B, I, O, and E to represent named entities, where B and E denote the beginning and end of the entity, I denotes the interior of the entity, and O denotes a non-entity. There are 19 other tag types in this dataset, including B-type, I-type, E-type, and O. The number of entity types is six. The initial label is $\varvec{I}$=($\varvec{i}_1$,$\varvec{i}_2$,...$\varvec{i}_n$). The label sequence is encoded using random initialization embedding to obtain the label matrix $\varvec{L}$=($\varvec{l}_{B-FRU}$,$\varvec{l}_{I-FRU}$,$\varvec{l}_{E-FRU}$,...$\varvec{l}_{O}$), where $\varvec{l}_{B-FRU}$ denotes the vector representation corresponding to the label B-FRU. The calculation is defined as Eq. (5):
  5 $$\begin{aligned} L=W_l I+b_l \end{aligned}$$
  By looking up the label matrix, the output of CRF is processed as sequence $\varvec{V}$=($\varvec{v}_1$,$\varvec{v}_2$,...$\varvec{v}_n$), where $\varvec{v}_{i}$ is the label embedding of the $\hbox {i}_{th}$ character. Then a label vector is constructed based on the corresponding label of each entity.
- *Relation-object detection algorithm*
  **Binary tagger** The relation-object tagger consists of a set of binary taggers that detect the position of the start and end of the object and assign a binary tag of 0 and 1 to each character. When the input character is identified as the head or tail of an object, the binary tagger is marked as 1. In other cases, it is marked as 0. Finally, a span representation of the object is constructed according to the proximity principle.
  **Object detection** The character embedding $\varvec{x}_{i}$, the label embedding of subject $\varvec{l}_{sub}^k$, and the label embedding of character $\varvec{v}_{i}$ are summed and entered into the relation-object recognition model, and the relation-object tagger calculates the probabilities for each character are defined as Eqs. (6) and (7):
  6 $$\begin{aligned} p_i^{start}= & {} \sigma (W_{start}^r(x_i+l_{sub}^k+v_i)+b_{start}^r)\end{aligned}$$
  7 $$\begin{aligned} p_i^{end}= & {} \sigma (W_{start}^r(x_i+l_{sub}^k+v_i)+b_{start}^r) \end{aligned}$$
  where $\hbox {p}_{i}^{start}$ denotes the probability that the $\hbox {i}_{{th}}$ character in the input sequence is the starting index position; $\hbox {p}_{i}^{end}$ denotes the probability that the $\hbox {i}_{{th}}$ character in the input sequence is the ending index position. $\varvec{W}_{start}^r$ and $\varvec{b}_{start}^r$ are the relation model parameters; k represents the number of subjects in the input sequence; $\varvec{l}_{sub}^k$ denotes the label vector of the $k_{th}$ subject. When $\hbox {p}_{i}^{start}$ is larger than threshold, the index position is set to 1, representing the starting position of the input character as the object, and $\hbox {p}_{i}^{end}$ is the same as $\hbox {p}_{i}^{start}$. Using the nearest start/end pair matching principle, the corresponding object is derived according to 1. That is, the model can construct a triplet of the subject, the relation, and its object.
  As shown in the Fig. 1, the word “Shaanxi” of the input sequence, and its character vector is represented as $\varvec{x}_{9}$; its label embedding is $\varvec{v}_{9}$. The first subject “kiwifruit” corresponds to the relation model input, which should be $\varvec{x}_{9}$+$\varvec{l}_{sub}^1$ +$\varvec{v}_{9}$, where $\varvec{l}_{sub}^1$=$\varvec{l}_{B-FRU}$+$\varvec{l}_{I-FRU}$+$\varvec{l}_{E-FRU}$.Then, according to the predefined relations in the text corpus:“process”, “contain”, “origin”, etc., the object is evaluated by the relation-object model, and the result is that “Shaanxi” is the begin and end character of the object corresponding to the subject “kiwifruit” under the relation “process” and “origin”. The final output are the triplets: (kiwifruit,process,Shaanxi), (kiwifruit,origin,Shaanxi).
- Loss Function
  The total loss of the model is the sum of the losses of each extraction section under the predefined relation, and the parameters in the optimization model are trained by minimizing the total loss. We use the cross-entropy loss for SOOT in Eq. (8):
  8 $$\begin{aligned} Loss=\textstyle \sum _{r\mid {j\in (s,o)}}y_i^jlogp_i^j+(1-y_i^j)(1-logp_i^j) \end{aligned}$$
  where j denotes s (subject) or o (object), $\hbox {y}_{i}^j$ represents the true value of the $\hbox {i}_{{th}}$ entity and $\hbox {p}_{i}^j$ represents the predicted value of the ith entity.

## Experiments

**Figure 2:**
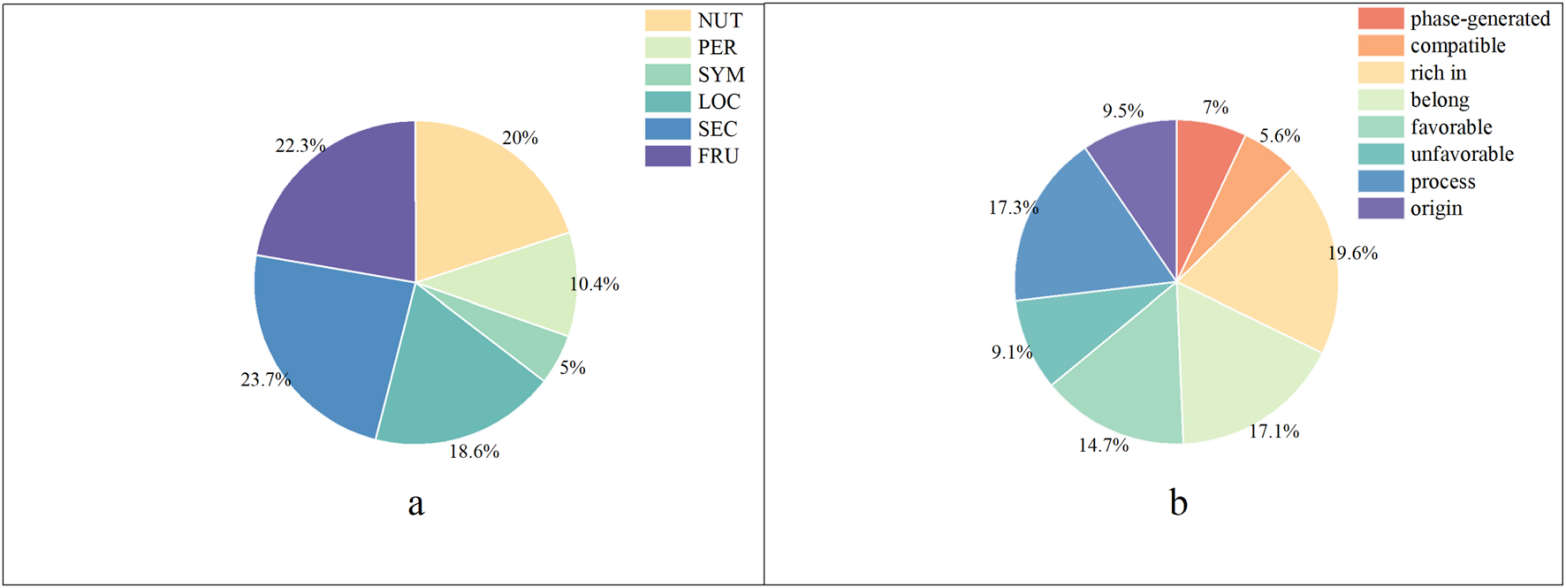
(**a**), (**b**) are the type distribution of entity and relation separately.

**Figure 3:**
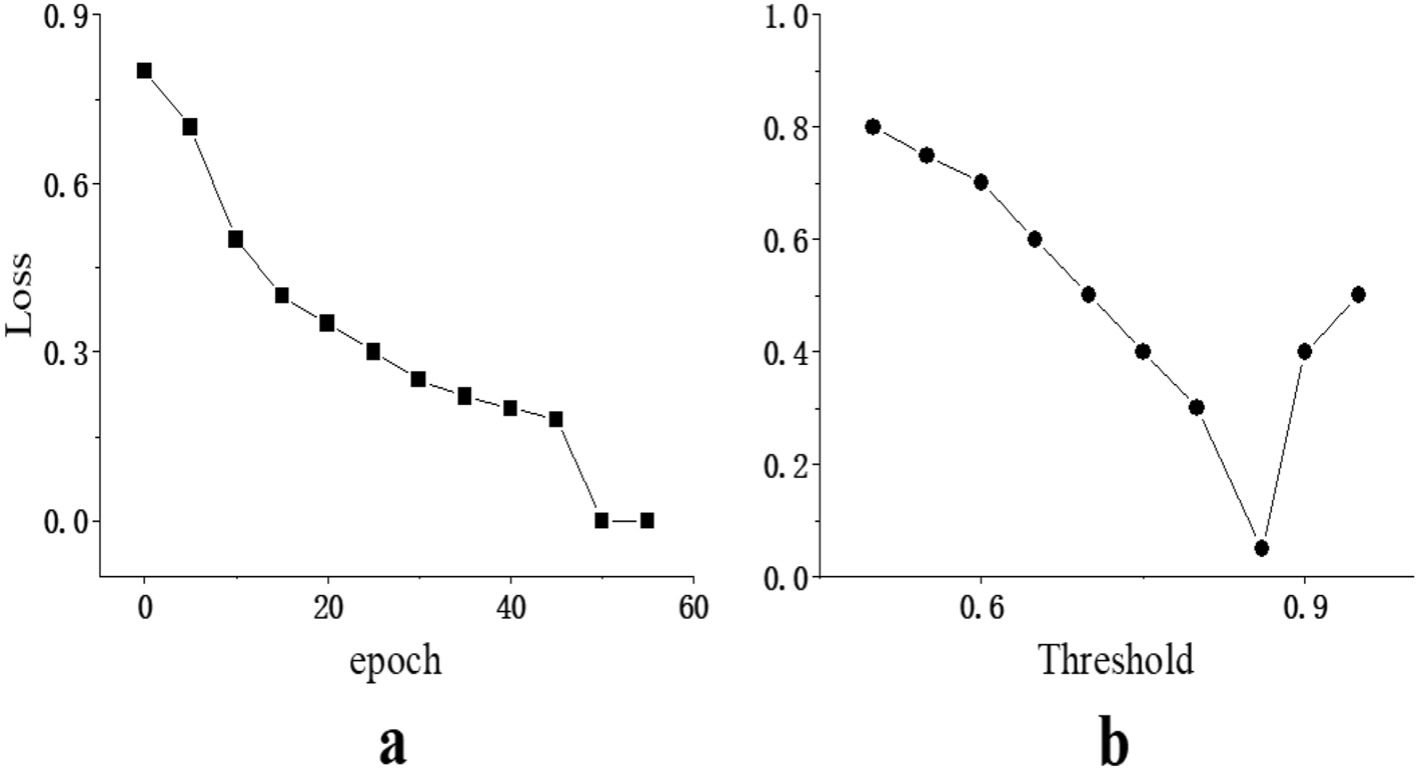
Determination of different parameters.

- *Dataset*
  The purpose of this study is to design a model with high performance and extraction accuracy to automatically extract triplets in the field of fruit nutrition. A fruit nutrition domain self-built dataset (FND) is constructed by crawling fruit nutrition information from official websites and other websites using Python crawlers. The fruit text corpus is split into 12,391 sentences. For data annotation in this study, we use the corpus annotation system developed by the National Engineering Laboratory of Agricultural Products Quality Tracking Technology and Applications. The FND contains 133 fruit species and 13,502 triplets, and more than 35% are overlapping triplets. By mining the text information, we predefine six entity classes: FRU(fruit), SEC (science), NUT (nutrition), PER (person), SYM (symptom), and LOC (location), and eight relation categories: belong, rich in, favorable, unfavorable, phase-generated, compatible, origin, and process location. The distribution of the FND is shown in Fig. 2.
- *Experimental setup*
  In this study, we determine the size of the hyper-parameters by conducting experiments using a validation set. The pre-trained language model we introduced is [BERT-wwm-ext, Chinese], and the model is trained in a GPU environment using the Pytorch framework. The FND is partitioned into a training set, a validation set, and a test set at a ratio of 7:2:1. The parameter settings are listed in Table 2 for all model experiments.
  Fig. 3a shows the changes of the loss; the model effect tends to gradually stabilize with increasing epoch. The F1 value reaches its highest value at the 50th training epoch, and we determine the epoch to be 50. Fig. 3b shows the variation of loss with threshold. The loss reaches a minimum when the threshold is 0.86, therefore, the threshold is set to 0.86.
- *Evaluation metrics*
  To verify the validity of SOOT and evaluate the performance of the experiment, we adopt precision, recall, and F1 as primary evaluation metrics. When a subject is recognized, the object should be detected under a predefined relation. Moreover, the subject and object are regarded as correct when their content, label, and boundary are all identified correctly.
- *Baseline model*
  In this study, we compare our model with four strong state-of-the-art models, namely, PA-LSTM-CRF^15^, CopyRE^29^, CASREL^30^, and BERT-JEORE^28^. The experiments are conducted on the publicly available NYT dataset as well as the self-built dataset (FND). NYT is one of the main English datasets utilized for overlapping triplet extraction tasks in recent years.
  Four models are applied for the comparison experiments: (1) PA-LSTM-CRF added position awareness, which required a position calculation for each character of the input sentence sequence and captured the position information of each query subject. (2) CopyRE consisted of an encoder and a decoder. The encoder converted natural language text into a fixed-length semantic vector, and the decoder generated a triplet from this semantic vector. (3) CASREL designed subject tagger and relation-specific object tagger to extract overlapping triplets. It performed remarkably well on NYT. (4) BERT-JEORE designed a three-step process for the extraction of overlapping issues by BERT to obtain contextual information shared to two downstream subtasks, and then extracted entities and relations by applying a source-target BERT model.
- *Analysis of experimental results*
  **Main results of comparison experiments** As shown in Table 3, SOOT outperforms the baseline models for three evaluation metrics on both the NYT and FND datasets, and the F1 value of SOOT is higher than the best baseline model by 4.8% and 2.5%, respectively.
  Unlike CASREL, PA-LSTM-CRF, and BERT-JEORE, SOOT tries BERT and CRF to extract entities, which can adequately obtain their position and label features and improve extraction accuracy. Comparing SOOT with CopyRE, the results show that modeling the relation as a subject-to-object mapping function can improve the efficiency of extracting overlapping triplets. Fusion label features in the base model enrich the input information of the relation evaluation module and improve the accuracy and F1 value of the extraction task. The analysis indicates that the above results are caused by (1) the subject module of this study considering the position features and contextual information of entities in sentences and (2) the hybrid label information of the subject module being integrated into the relation model, improving the subject-object correspondence in a specific relation.
  **Ablation Test** To further analyze the effectiveness of each component of SOOT, we design several additional experiments. First, we replace BERT with word2vec, resulting in the F1 value dropping by 3.3%, Nonetheless, $\hbox {SOOT}_{{Word2Vec}}$ is still competitive against existing state-of-the-art models. This validates the utility of the proposed SOOT model.We also try LSTM as the encoder for the entire model, but the performance does not well. As the current input token of LSTM is closely related to the previous input token, it does not perform well for long sequences. As the length of the sequence increases, the effect of distant and important information on the currently entered token is weakened. BERT solves this problem by applying position encoding and Attention mechanism. Next, we add the Bi-LSTM above the BERT layer, and the performance of subject recognition improves slightly. This means that CRF could capture the label information fairly well. Finally, when we remove the subject label embedding from the input of the relation-object detection model, the performance of the model drops significantly. Similarly, the F1 value of the model is reduced when we remove the character vector. This indicates that each label embedding process plays a vital role in object detection. Table 4 shows the performance of the ablation test.

**Table 2:** Parameter settings.

Parameters	Value
BatchSize	32
Sentence_Maxlenth	100
Threshold	0.86
Dropout	0.2
Optimzier	Adam

**Table 3:** Comparison of experimental results.

Model	FND			NYT		
	P	R	F1	P	R	F1
PA-LSTM-CRF	0.434	0.544	0.483	0.494	0.591	0.538
CopyRE	0.779	0.672	0.721	0.732	0.645	0.686
BERT-JEORE	0.84	0.884	0.864	0.885	0.864	0.865
CASREL	0.837	0.884	0.859	0.897	0.895	0.896
$\hbox {SOOT}_{-sl}$	0.84	0.884	0.861	0.898	0.896	0.897
$\hbox {SOOT}_{-cl}$	0.915	0.893	0.904	0.92	0.918	0.919
**SOOT**	**0.915**	**0.9**	**0.907**	**0.923**	**0.919**	**0.921**

Significant values are in [bold].

**Table 4:** Feature settings.

Feature	F1
**SOOT**	**0.907**
Word2Vec	0.874($\downarrow$)
LSTM	0.889($\downarrow$)
+ Bi-LSTM	0.909($\rightarrow$)
- character label embedding	0.904($\downarrow$)
-subject label embedding	0.861($\downarrow$)

Significant values are in [bold].

## Discussion

In this section, we elaborate on the reasons behind the impressive performance of the model

**Figure 4:**
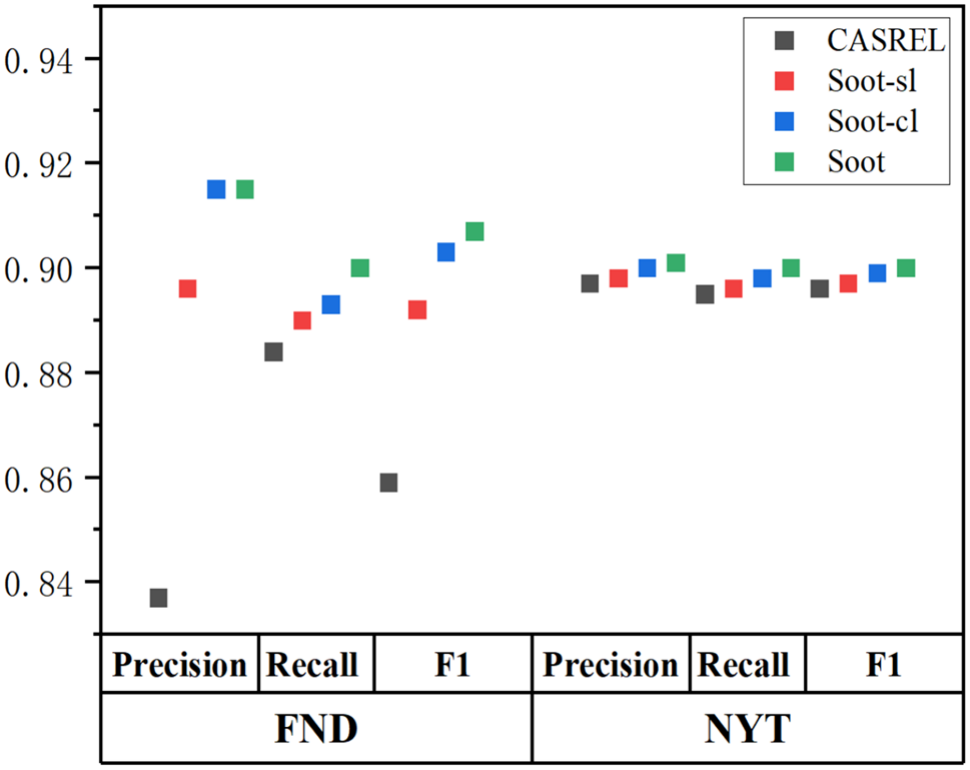
The comparison of different versions of SOOT with CASREL.

**Figure 5:**
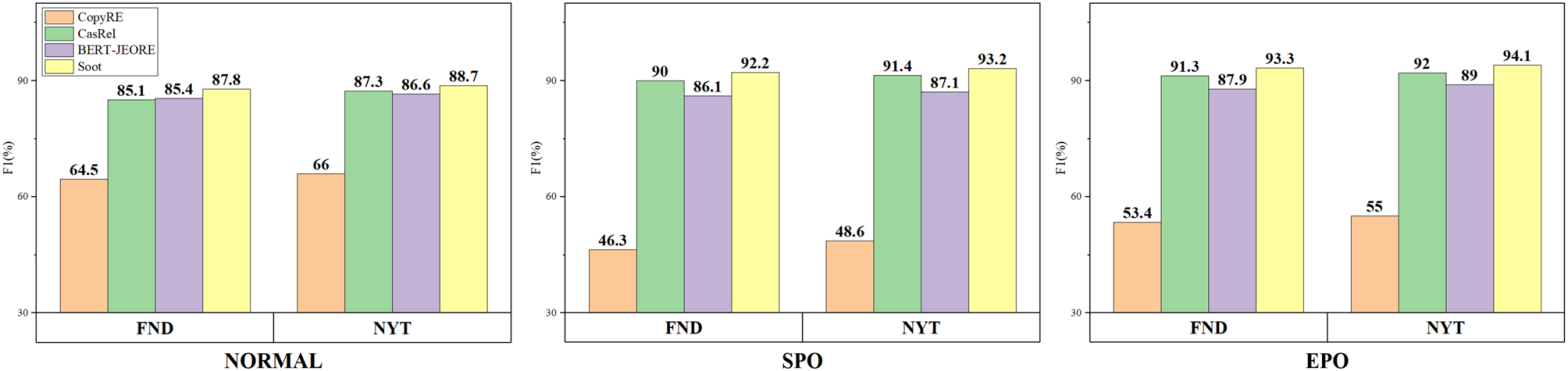
F1 values on different triplet types.

- *Hybrid label embedding*
  This paper considers entity features, including content, boundary, label and position information. $\hbox {SOOT}_{-sl}$ only retains the character feature, and it significantly outperforms the basic models using the FND. $\hbox {SOOT}_{-cl}$ removes the character label feature from SOOT and retains the subject label. $\hbox {SOOT}_{-cl}$ increases both the precision and F1 value 2.3% compared with the base model using the NYT. This demonstrates that only applying label feature can effectively improve the performance of the model. SOOT integrates hybrid label information into the overlapping triplet extraction and achieves F1 value improvements of 4.8% and 2.5% for FND and NYT, respectively. Fig. 4 shows the comparison between the different versions of SOOT and CASREL.
- *Model function*
  Normally, the problem of triplet extraction is transformed into a classification problem by first listing all entity pairs, and then using a classifier to detect the relation. Nonetheless, the multiple relations between entity pairs cannot be easily recognized in a general way. To overcome the overlapping problem of triplet extraction, SOOT models the relation as a function of the subject mapped to the object by adopting the subject and relation to identify the object. Therefore, it can easily handle situations in which one subject corresponds to multiple objects. To verify the power of the SOOT model in dealing with the overlapping problem, we perform further experiments using the FND and NYT datasets. In Fig. 5, the F1 values of the comparison model and SOOT for different triplet types show that SOOT outperform the comparison model for all triplet types.

## Conclusions

In this paper, we propose a subject-oriented overlapping triplet extraction model for solving the overlapping problem in the fruit nutrition knowledge corpus. This model divides the task into two sub-algorithms. The subject algorithm is applied to predict all possible entities and their corresponding labels in a sentence. The relation-object algorithm is introduced to detect the object corresponding to a given subject via a specific relation. Experiments on Chinese self-constructed and public English datasets show that the subject-oriented method can improve the efficiency of extracting overlapping triplets.The proposed work can effectively mine agricultural knowledge from complex agricultural data and builds a knowledge base in the field of fruit nutrition. Furthermore, it can also facilitate the development of intelligent recommendation and intelligent Q &A applications for food nutrition matching. In our future work, we will further investigate the English corpus in the field of fruit nutrition to explore the performance of our model on the English fruit nutrition dataset.

## Data Availability

The data used to support the findings of this study are available at https://gitee.com/malei0506/fnd/tree/master/.
